# Modeling the Release Kinetics of Poorly Water-Soluble Drug Molecules from Liposomal Nanocarriers

**DOI:** 10.1155/2011/376548

**Published:** 2011-06-07

**Authors:** Stephan Loew, Alfred Fahr, Sylvio May

**Affiliations:** ^1^Department of Physics, North Dakota State University, Fargo, ND 58108-6050, USA; ^2^Department of Pharmaceutical Technology, Friedrich Schiller University of Jena, Lessingstraße 8, 07743 Jena, Germany

## Abstract

Liposomes are frequently used as pharmaceutical nanocarriers to deliver poorly water-soluble drugs such as temoporfin, cyclosporine A, amphotericin B, and paclitaxel to their target site. Optimal drug delivery depends on understanding the release kinetics of the drug molecules from the host liposomes during the journey to the target site and at the target site. Transfer of drugs in model systems consisting of donor liposomes and acceptor liposomes is known from experimental work to typically exhibit a first-order kinetics with a simple exponential behavior. In some cases, a fast component in the initial transfer is present, in other cases the transfer is sigmoidal. We present and analyze a theoretical model for the transfer that accounts for two physical mechanisms, collisions between liposomes and diffusion of the drug molecules through the aqueous phase. Starting with the detailed distribution of drug molecules among the individual liposomes, we specify the conditions that lead to an apparent first-order kinetic behavior. We also discuss possible implications on the transfer kinetics of (1) high drug loading of donor liposomes, (2) attractive interactions between drug molecules within the liposomes, and (3) slow transfer of drugs between the inner and outer leaflets of the liposomes.

## 1. Introduction

Poor solubility in water is a well-recognized obstacle for efficient oral or parenteral drug administration [1, 2]. Liposomes are among the most widely used type of pharmaceutical nanocarriers for small and poorly water-soluble drug molecules [3]. These drugs preferentially partition into the hydrophobic compartment that is formed by the hydrocarbon tails of the liposomal lipids. Liposomes have been used in their first generation (conventional liposomes) predominantly as long-circulating transport vehicles [4, 5], followed by a second generation that improved the circulation time further by decorating the surface with PEG-chains (stealth liposomes [6]). Third-generation liposomes are now being engineered to contain targeting ligands [7] and to carry out stimuli-sensitive triggering of the drug release [8].

An important property of liposome-based drug delivery is the release kinetics of the drug from the host, which has been investigated for a number of model systems [9–12]. Experimental investigations of the transfer of temoporfin between two different types of liposomes (i.e., from donor liposomes to acceptor liposomes) have recently been carried out using a mini ion exchange column technique [13]. The column separates donor from acceptor liposomes and thus allows to monitor the time dependence of the drug transfer. It is observed that, typically, the transfer follows an apparent first-order behavior, characterized by a single exponential function. This is remarkable given the complexity of the system, with the drug molecules being able to migrate from the donor to the acceptor liposomes via different physical mechanisms. In fact, there are two mechanisms that, in general, act simultaneously. The first mechanism is the transfer of drugs upon collisions between two liposomes. In this case, the drug molecules directly migrate from one liposome to another with minimal exposure to the aqueous phase. The second mechanism refers to the transfer of drugs via diffusion through the aqueous phase. We note that the collision mechanism has been invoked, for example, to explain the transfer of lipids [14] and cholesterol [15] between vesicles, and the transfer of fatty acids between vesicles and fatty acid binding proteins [16]. Also the diffusion mechanism was found to be consistent with the transport of lipids [17]. In some cases, both mechanisms were suggested to contribute to the transport of lipids between vesicles [18] and to the transport of lipophilic drugs from oil-in-water emulsions to cells [19] and from plasma proteins to lipid vesicles [20]. In our preceding experimental work, where we have investigated the kinetics of temoporfin transport from donor to acceptor liposomes [13], we found that above a certain concentration (corresponding to a liposome-to-liposome distance of about 200 nm for our specific system) the transfer was dominated by collisions; for smaller concentrations transport through diffusion was prevalent.

The objective of the present work is to introduce and discuss a detailed kinetic model for the release properties of poorly water-soluble drug molecules from liposomal nanocarriers. Despite a large number of experimental studies about the kinetics of lipid and drug transfer between liposomes and other nanocarriers, there is little theoretical work available that addresses the nature of the transfer kinetics. Our theoretical formalism is based on a detailed distribution function of drug molecules among the individual liposomes. Kinetic rate equations for that distribution function account for two transport mechanisms: collisions between liposomes and drug diffusion through the aqueous phase. We specify a set of conditions at which our microscopic model produces an apparent first-order kinetics with simple exponential behavior, as used in previous work [14, 19]. We point out that our kinetic model can be applied to any kind of small mobile pharmaceutical nanocarrier, including liposomes, micelles [21], colloids [22], and nanoparticles [23].

In the second part of our work, we discuss conditions that lead to deviations from simple exponential behavior. First, for the diffusion mechanism, high drug loading tends to increase the transfer rate. The kinetics remains exponential only if donor and acceptor liposomes are chemically similar. Second, the presence of attractive interactions between drug molecules within the liposomes (which can result in the formation of aggregates [24]) is expected to slow down the transfer kinetics. We note that not much molecular detail is presently known about how poorly water-soluble drug molecules inside a lipid bilayer interact. However, modeling studies of rigid membrane-embedded inclusions such as transmembrane proteins or peptides suggest a * general* tendency of the host membrane to mediate attractive interactions between inclusions that may lead to the formation of aggregates [25]. These attractive interactions may be driven by elastic deformations of the host membrane [26], by depletion of the flexible lipid chains from the region in between rigid inclusions [27], and by fluctuations via the Casimir effect [28]. Our analysis for the collision mechanism suggests that aggregate formation can give rise to sigmoidal behavior of the transfer kinetics. Third, drug molecules (even if they are poorly water soluble) do not necessarily reside predominantly in the innermost region of the membrane's hydrocarbon region. For example, some aromatic compounds such as indole are well known for their preference of the membrane's interfacial region between the headgroups and the hydrocarbon chains [29, 30]. Other aromatic compounds such as benzene are distributed throughout the hydrocarbon chain region without a bias toward the polar/apolar interface [30]. Among the reasons for the preferential partitioning of indole are electrostatic interactions, hydrogen bond formation, and the steric shape of the molecule. For lipid monolayers, there is evidence that drug partitioning also depends on the lateral pressure [31]. Generally, whenever a drug molecule interacts more favorably with the interfacial or headgroup region than with the hydrocarbon tail region, the corresponding partitioning preference can be lumped into at least two energetically preferred states that correspond to the inner and outer leaflet of the membrane. Transfer between the two states (i.e., flip-flop) then introduces an additional characteristic time [32]. We note that two- or multiple-state modeling has been invoked previously to model the partitioning of amino-acid analogues in membranes [33] and the permeation of drug molecules through membranes [34, 35].

## 2. Model

Consider an aqueous solution (of fixed volume *V*) that contains a number of *N*_*d*_ donor and *N*_*a*_ acceptor liposomes. Donor and acceptor liposomes may be either two chemically different types of liposomes (i.e., composed of different lipids) or equivalent liposomes (i.e., containing the same lipid composition). In the latter case, the distinction between donor and acceptor liposomes refers only to their initial loading; at the end of the transport process (i.e., at thermal equilibrium), both types would be indistinguishable.

The donor liposomes initially carry a total number of *M* poorly water-soluble drug molecules. Transfer of drug molecules from donor to acceptor liposomes will take place with time until, eventually, an equilibrium partitioning is reached. We describe the time dependence of this transfer by the number of drug molecules carried by the donor liposomes, *M*_*d*_(*t*), and by the acceptor liposomes, *M*_*a*_(*t*). It is then initially *M*_*d*_(*t* = 0) = *M* and *M*_*a*_(*t* = 0) = 0, as well as in equilibrium *M*_*d*_(*t* → *∞*) = *M*_*d*_^eq^ and *M*_*a*_(*t* → *∞*) = *M*_*a*_^eq^, where *M*_*d*_^eq^ and *M*_*a*_^eq^ denote the equilibrium number of drug molecules carried by donor and acceptor liposomes, respectively. We point out that, although we refer to the drug carriers as liposomes, our model is more general. That is, it can be applied to different types of mobile carriers such as micelles, colloids, nanoparticles, or polymeric aggregates, given the carrier possesses some capacity to host poorly water-soluble drug molecules.

**Figure 1:**
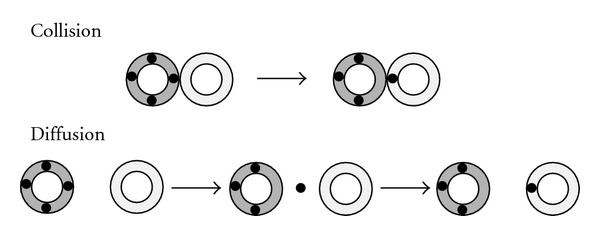
Transfer of a drug molecule (black bullets) from donor liposome (dark-shaded) to acceptor liposome (light-shaded) upon the collision of the two liposomes or upon diffusion of the drug molecule through the aqueous phase. The displayed scheme refers to the special situation of initially empty acceptor liposomes but analogous schemes apply to any other initial situation.

In the following, we suggest a model for the time dependence of the transfer process (i.e., for *M*_*d*_(*t*) and *M*_*a*_(*t*)) that leads to a first-order kinetics, characterized by a simple exponential function. We consider a “single-state model” where there is only a single energetic state available for each drug molecule in a given liposome. The single-state model excludes the presence of intraliposomal kinetics (the extension to a two-state model will be discussed below). We account for two different transport mechanisms: (i) transport through collisions between liposomes and (ii) transport via diffusion of drug molecules through the aqueous phase. Both mechanisms are schematically illustrated in Figure 1.

Our transport model of drugs from donor to acceptor liposomes employs the framework of chemical reaction kinetics. We note that due to the generally slow release kinetics of poorly water-soluble drugs, we can treat the aqueous solution as *spatially uniform* at all times. Hence, no combined diffusion-reaction kinetics [36] needs to be included in our model.

### 2.1. Transfer through Collisions Only

Our model for the collision-mediated drug transfer between liposomes starts with the detailed distribution of drug molecules among all liposomes. We introduce the number *d*_*j*_ of donor liposomes that carry *j* drug molecules. An analogous definition is used for the number *a*_*j*_ of acceptor liposomes that carry *j* drug molecules. The index *j* is confined to the region 0 ≤ *j* ≤ *m* where *m* is the maximal number of drug molecules that a liposome can carry. The time-dependent distribution functions *d*_*j*_ = *d*_*j*_(*t*) and *a*_*j*_ = *a*_*j*_(*t*) represent a full microscopic knowledge of the kinetics of drug transfer. The total numbers of donor liposomes *N*_*d*_, acceptor liposomes *N*_*a*_, drug molecules residing in donor liposomes *M*_*d*_, and drug molecules residing in acceptor liposomes *M*_*a*_, can be calculated on the basis of the distribution functions *d*_*j*_ = *d*_*j*_(*t*) and *a*_*j*_ = *a*_*j*_(*t*) according to

(1) $$\begin{array}{c} N_{d}\;=\overset{m}{\underset{j=0}{\sum }}d_{j},\;\;N_{a}=\overset{m}{\underset{j=0}{\sum }}a_{j},\\ M_{d}=\overset{m}{\underset{j=0}{\sum }}jd_{j},\;\;M_{a}=\overset{m}{\underset{j=0}{\sum }}ja_{j}. \end{array}$$

**Figure 2:**
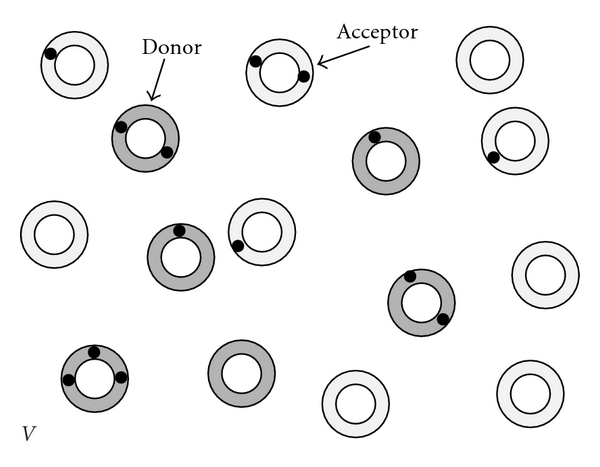
Exemplification of our system: *N*_*d*_ = 6 donor liposomes (dark shaded) and *N*_*a*_ = 9 acceptor liposomes (light shaded) reside in an aqueous space of volume *V*; each liposome can carry at most *m* = 3 drug molecules (black bullets). For the displayed hypothetical snapshot (taken at a certain time *t*), the distribution function of drug molecules among the donor liposomes is *d*_0_ = 1, *d*_1_ = 2, *d*_2_ = 2, *d*_3_ = 1, leading to a total of *M*_*d*_ = ∑_*j*=0_^*m*^ *jd*_*j*_ = 9 drug molecules residing in donor liposomes. Analogously for the acceptor liposomes, the distribution function is *a*_0_ = 5, *a*_1_ = 3, *a*_2_ = 1, *a*_3_ = 0, implying *M*_*a*_ = ∑_*j*=0_^*m*^ *ja*_*j*_ = 5.

Mathematically, *N*_*d*_ and *N*_*a*_ are the zeroth-moments of the distributions functions *d*_*j*_ = *d*_*j*_(*t*) and *a*_*j*_ = *a*_*j*_(*t*) whereas *M*_*d*_ and *M*_*a*_ appear as the corresponding first moments. We assume that *N*_*d*_ and *N*_*a*_ are constant (i.e., independent of time), and so then is the total number of liposomes *N* = *N*_*d*_ + *N*_*a*_. This is appropriate if fusion and fission between liposomes can be ignored. Due to our focus on poorly water-soluble drug molecules, it is also justified to assume that the total number of drug molecules carried by all liposomes, *M* = *M*_*d*_ + *M*_*a*_, is constant. That is, we neglect the small fraction of drug molecules that reside in the aqueous phase without being bound to a liposome.  Figure 2 schematically illustrates a specific exemplification of the system. 

**Figure 3:**
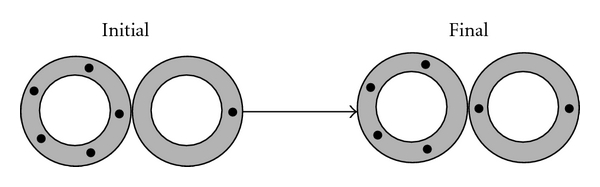
Transfer of a drug molecule (black bullets) upon the collision of two liposomes (here assumed to be two donor liposomes). The drug distribution function changes from initially *d*_1_ = 1, *d*_2_ = 0, *d*_3_ = 0, *d*_4_ = 0, *d*_5_ = 1 to *d*_1_ = 0, *d*_2_ = 1, *d*_3_ = 0, *d*_4_ = 1, *d*_5_ = 0. This represents an example (for *i* = 5 and *j* = 1) of the general scheme *d*_*i*_ → *d*_*i*_ − 1, *d*_*i*−1_ → *d*_*i*−1_ + 1, *d*_*j*_ → *d*_*j*_ − 1, and *d*_*j*+1_ → *d*_*j*+1_ + 1.

Collisions require two liposomes to come to close proximity. The magnitude of drug transport between, say, donor liposomes *d*_*i*_ and *d*_*j*_ is thus ~*d*_*i*_ × *d*_*j*_/*V* where *V* is the volume of the aqueous solution. The underlying transfer process is thus second order. If a single drug molecule is transferred from a donor that carries initially *i* drug molecules to a donor that carries initially *j* drug molecules, the distribution function changes according to *d*_*i*_ → *d*_*i*_ − 1, *d*_*i*−1_ → *d*_*i*−1_ + 1, *d*_*j*_ → *d*_*j*_ − 1, and *d*_*j*+1_ → *d*_*j*+1_ + 1. Hence, the numbers *d*_*i*_ and *d*_*j*_ decrease whereas *d*_*i*−1_ and *d*_*j*+1_ increase.  Figure 3 shows an illustration of this scheme for *i* = 5 and *j* = 1. The transfer rate between the populations *d*_*i*_ and *d*_*j*_ will also depend on the corresponding numbers of drug molecules *i* and *j*. We assume the drug molecules within each liposome form an ideal mixture so that the transfer rate is directly proportional to |*i* − *j*|. In writing a rate equation for donor population *d*_*j*_, we have to account for all possible ways of collisions between donor liposomes of index *j* with other liposomes (donors and acceptors) of index *i*. These considerations lead us to

(2) $$\begin{array}{cc} \frac{V}{K_{\text{coll}}}{\dot{d}}_{j} & =\overset{j}{\underset{i=0}{\sum }}d_{i}\left[d_{j+1}g\left(j+1,i\right)-d_{j}g\left(j,i\right)\right]\\ & \;\;\;\;+\overset{m}{\underset{i=j}{\sum }}d_{i}\left[d_{j-1}g\left(i,j-1\right)-d_{j}g\left(i,j\right)\right]\\ & \;\;\;\;+\overset{j}{\underset{i=k}{\sum }}a_{i-k}\left[d_{j+1}g\left(j+1,i\right)-d_{j}g\left(j,i\right)\right]\\ & \;\;\;\;+\overset{m+k}{\underset{i=j}{\sum }}a_{i-k}\left[d_{j-1}g\left(i,j-1\right)-d_{j}g\left(i,j\right)\right], \end{array}$$

where we have defined the function

(3) $$\begin{array}{c} g\left(i,j\right)=i-j. \end{array}$$

In (2), *K*_coll_ is the unit rate of drug transfer through collisions between two chemically equivalent liposomes, and $\dot{x}=dx/dt$ denotes the time derivative of a physical quantity *x*(*t*). The first two lines in (2) account for collisions of donor liposomes with other donor liposomes. The last two lines in (2) account for collisions of donor liposomes with acceptor liposomes.

Note that (2) allows for a difference in the chemical nature of donor and acceptor liposomes. This chemical mismatch is accounted for by the integer *k* in the last two lines of (2), which expresses the difference in the number of drug molecules between a donor and acceptor liposomes in thermal equilibrium, (i.e., for *k* = 0 each donor and acceptor liposome will contain the same number of drug molecules in thermal equilibrium). We do not attempt to calculate *k* from a microscopic model; yet below we show how *k* is related to the change in standard Gibbs free energy for the process of transferring drug molecules from donor to acceptor liposomes.

Due to symmetry, we obtain ${\dot{a}}_{j}$ from ${\dot{d}}_{j}$ by replacing *d*_*i*_ → *a*_*i*_, *a*_*i*_ → *d*_*i*_, and *k* → −*k*,

(4) $$\begin{array}{cc} \frac{V}{K_{\text{coll}}}{\dot{a}}_{j} & =\overset{j}{\underset{i=0}{\sum }}a_{i}\left[a_{j+1}g\left(j+1,i\right)-a_{j}g\left(j,i\right)\right]\\ & \;\;\;\;+\overset{m}{\underset{i=j}{\sum }}a_{i}\left[a_{j-1}g\left(i,j-1\right)-a_{j}g\left(i,j\right)\right]\\ & \;\;\;\;+\overset{j}{\underset{i=-k}{\sum }}d_{i+k}\left[a_{j+1}g\left(j+1,i\right)-a_{j}g\left(j,i\right)\right]\\ & \;\;\;\;+\overset{m-k}{\underset{i=j}{\sum }}d_{i+k}\left[a_{j-1}\;g\left(i,j-1\right)-a_{j}g\left(i,j\right)\right]. \end{array}$$

Equations (2) and (4) constitute a microscopic model for the kinetic behavior of drug transport from donor to acceptor liposomes through the collision mechanism; it can be verified that

(5) $$\begin{array}{c} \overset{m}{\underset{j=0}{\sum }}{\dot{d}}_{j}=\overset{m}{\underset{j=0}{\sum }}{\dot{a}}_{j}=\overset{m}{\underset{j=0}{\sum }}j\left({\dot{a}}_{j}+{\dot{d}}_{j}\right)=0, \end{array}$$

implying ${\dot{N}}_{d}={\dot{N}}_{a}=\dot{M}=0$ and thus ensuring conservation of the number of donor and acceptor liposomes (*N*_*d*_ and *N*_*a*_) as well as of the total number of drug molecules (*M* = *M*_*d*_ + *M*_*a*_). To characterize the total numbers *M*_*d*_ and *M*_*a*_ of drug molecules that reside in donor and acceptor liposomes, respectively, we carry out the summations $\sum_{j=0}^{m}j{\dot{d}}_{j}$ and $\sum_{j=0}^{m}j{\dot{a}}_{j}$ using (2) and (4). The result are the two first-order differential equations

(6) $$\begin{array}{cc} {\dot{M}}_{d} & =\frac{K}{N}\left(M_{a}N_{d}-M_{d}N_{a}+kN_{a}N_{d}\right),\\ {\dot{M}}_{a} & =\frac{K}{N}\left(M_{d}N_{a}-M_{a}N_{d}-kN_{a}N_{d}\right), \end{array}$$

where we have introduced the definition of the apparent rate constant

(7) $$\begin{array}{c} K=K_{\text{coll}}\;\frac{N}{V}. \end{array}$$

Initially, all drug molecules are incorporated in the donor liposomes, implying *M*_*d*_(*t* = 0) = *M* and *M*_*a*_(*t* = 0) = 0. The solution of (6) is then

(8) $$\begin{array}{c} M_{a}\left(t\right)=M-M_{d}\left(t\right)=\left(1-e^{-Kt}\right)\frac{N_{a}}{N}\;\left(M-kN_{d}\right). \end{array}$$

Hence, *K* indeed appears as the inverse characteristic time for the transfer process. In contrast to previous models [14], *K* depends only on the total concentration of liposomes *N*/*V* but not on the concentrations of donor or acceptor liposomes individually. We also mention that (6) and the solution in (8) are valid for any number of donor and acceptor liposomes (i.e, any choice of *N*_*d*_ and *N*_*a*_). This includes but is not restricted to sink conditions (where *N*_*a*_ ≫ *N*_*d*_).

Thermodynamic equilibrium corresponds to the long-time limit, *t* → *∞*, at which we have *M*_*d*_ = *M*_*d*_^eq^ and *M*_*a*_ = *M*_*a*_^eq^ with

(9) $$\begin{array}{c} \frac{M_{d}^{\text{eq}}}{M}=\frac{N_{d}}{N}\left(1+k\frac{N_{a}}{M}\right),\;\frac{M_{a}^{\text{eq}}}{M}=\frac{N_{a}}{N}\left(1-k\frac{N_{d}}{M}\right). \end{array}$$

From (9), we obtain the difference between the numbers of drug molecules per donor and acceptor liposome, (*M*_*d*_^eq^/*N*_*d*_)−(*M*_*a*_^eq^/*N*_*a*_) = *k*. This agrees with our interpretation of *k* in (2) and (4). We note that for chemically identical donor and acceptor liposomes, it is *k* = 0 and all liposomes carry the same number of drug molecules in equilibrium, implying *M*_*d*_^eq^/*N*_*d*_ = *M*_*a*_^eq^/*N*_*a*_ = *M*/*N*. The largest possible value of *k* is *k* = *M*/*N*_*d*_ for which we obtain *M*_*a*_^eq^ = 0 and *M*_*d*_^eq^ = *M*. The smallest possible value of *k* is *k* = −*M*/*N*_*a*_ implying *M*_*a*_^eq^ = *M* and *M*_*d*_^eq^ = 0. Hence, −*M*/*N*_*a*_ ≤ *k* ≤ *M*/*N*_*d*_.

The solution in (8) corresponds to a simple exponential decay of the number of drug molecules in the donor liposomes. This suggests that we can express the transfer kinetics of drug molecules from donor (D) to acceptor (A) liposomes as the chemical reaction scheme

(10) $$\begin{array}{c} \text{D} \end{array}\overset{K_{1}}{\underset{K_{2}}{⇌}}\text{A},$$

with rate constants *K*_1_ and *K*_2_. The corresponding kinetic behavior is then governed by the equations ${\dot{M}}_{d}=-K_{1}M_{d}+K_{2}M_{a}$ and ${\dot{M}}_{a}=K_{1}M_{d}-K_{2}M_{a}$ where *M*_*d*_ = *M*_*d*_(*t*) and *M*_*a*_ = *M*_*a*_(*t*) are the numbers of drug molecules carried by donor and acceptor liposomes, respectively. With *M*_*d*_(*t* = 0) = *M* and *M*_*a*_(*t* = 0) = 0 we obtain

(11) $$\begin{array}{c} M_{a}\left(t\right)=M-M_{d}\left(t\right)=\left(1-e^{-(K_{1}+K_{2})t}\right)\left(\frac{K_{1}}{K_{1}+K_{2}}\right)\frac{N_{a}}{N}M \end{array},$$

which has indeed the same structure as (8). Comparison of (8) with (11) reveals *K*_1_ = (1 − *kN*_*d*_/*M*)*KN*_*a*_/*N* and *K*_2_ = (1 + *kN*_*a*_/*M*)*KN*_*d*_/*N*. The equilibrium constant *K*_*eq*_ = *K*_1_/*K*_2_ of the reaction in (10) is thus

(12) $$\begin{array}{c} K_{\text{eq}}=\frac{N_{a}}{N_{d}}\;\frac{M-kN_{d}}{M+kN_{a}}. \end{array}$$

Comparing this with *K*_eq_ = exp (−Δ*g* ^0^/*k*_*B*_ *T*) (where *k*_*B*_ is Boltzmann's constant and *T* is the absolute temperature) allows us to compute the change in standard Gibbs free energy

(13) $$\begin{array}{c} \Delta g^{0}=k_{B}T\ln\;\left(\frac{M/N_{a}+k}{M/N_{d}-k}\right), \end{array}\;$$

for the transfer of a single drug molecule from a donor to an acceptor liposome. The enthalpic and entropic contributions to Δ*g* ^0^ will be influenced by *k*, which is, generally, temperature dependent(*k* = *k*(*t*)). Let us briefly discuss two cases. First, if donor and acceptor liposomes are chemically identical, then *k* = 0 and Δ*g* ^0^ = *k*_*B*_ *T*ln (*N*_*d*_/*N*_*a*_) has only an entropic contribution. Specifically, for *N*_*d*_ > *N*_*a*_, we find Δ*g* ^0^ > 0 because a given drug molecule has more donor liposomes to reside in than acceptor liposomes. Second, the limiting cases for *k*, namely, *k* = −*M*/*N*_*a*_ and *k* = *M*/*N*_*d*_, yield Δ*g* ^0^ → −*∞* (thus, with all drugs migrating to the acceptor liposomes) and Δ*g* ^0^ → *∞* (thus with all drugs remaining in the donor liposomes), respectively.

We point out that our model predicts a simple exponential time behavior despite the presence of drug transfer through a second-order two-body collision process (i.e., collisions between two liposomes). Chemical reactions that deplete the reactants through binary collisions generally display a long time-tail *c*(*t*) ~ 1/*t* in their concentration dependence. For example, the kinetic behavior of the dimerization reaction 2 *monomer* → *dimer* follows the equation $\dot{c}=\overset{\sim }{k}c^{2}$ where *c*(*t*) is the concentration of the reactant (i.e., the monomers) and $\overset{\sim }{k}$ the rate constant. With an initial concentration *c*(*t* = 0) = *c*_0_ the time behavior becomes $c\left(t\right)=c_{0}/\left(1+\overset{\sim }{k}t\right)$, implying *c*(*t*) ~ 1/*t* for long times. For our system, however, the numbers of donor and acceptor liposomes remain unchanged. Thus, collisions do not deplete the reactants, and the concentration dependencies of *M*_*d*_(*t*) and *M*_*a*_(*t*) become exponential in time.

### 2.2. Transfer through Diffusion Only

Diffusion allows for transfer of drug molecules directly through the aqueous phase, without the need of collisions between liposomes. Denoting the additional state in the aqueous phase by W (in addition to donor (D) and acceptor (A)) the corresponding transport scheme (again, as in (10), formally expressed as a chemical reaction) can be written as [14, 37]

(14) $$\begin{array}{c} \text{D}\overset{K_{d}^{\mathrm{rel}\;}}{\underset{K_{d}^{\text{upt}}}{⇌}}\text{W}\overset{K_{a}^{\text{upt}}}{\underset{K_{a}^{\mathrm{rel}\;}}{⇌}}\text{A}, \end{array}\;\;$$

with rate constants *K*_*d*_^*rel*^, *K*_*a*_^*rel*^, *K*_*d*_^upt^, and *K*_*a*_^upt^ for the drug release (“rel”) and uptake (“upt”) in donor (“d”) and acceptor (“a”) liposomes. To formulate the rate equations, it is useful to first consider the drug distribution function *d*_*j*_(*t*). We assume the probability of a drug molecule to leave donor liposomes of index *j* to be proportional to the total number *jd*_*j*_ of drug molecules in that liposome population. Similarly, the probability of a drug molecule to enter donor liposomes of index *j* is assumed to be proportional to the total number (*m* − *j*)*d*_*j*_ of empty binding sites in that liposome population. Because the uptake is based on collisions of liposomes with drug molecules in the aqueous solution, the rate should also be proportional to the drug concentration *M*_*w*_/*V* in the aqueous phase (here, *M*_*w*_ is the total number of drug molecules residing in the aqueous phase). This leads to the following rate equations

(15) $$\begin{array}{cc} {\dot{d}}_{j} & =K_{d}^{\mathrm{rel}\;}\left[\left(j+1\right)d_{j+1}-jd_{j}\right]\\ & \;\;\;\;+K_{d}^{\text{upt}}\frac{M_{w}}{V}\left[\left(m-\left(j-1\right)\right)d_{j-1}-\left(m-j\right)d_{j}\right], \end{array}$$

for 0 ≤ *j* ≤ *m* (with *d*_*j*_ = *a*_*j*_ = 0 for *j* < 0 or *j* > *m*). A similar equation can be written for the acceptor liposomes. Based on (15), it can be verified that $\sum_{j=0}^{m}{\dot{d}}_{j}=0$, thus ensuring conservation of *N*_*d*_ (and similarly for *N*_*a*_). Carrying out the summation ${\dot{M}}_{d}=\sum_{j=0}^{m}j{\dot{d}}_{j}$ using (15) leads to

(16) $$\begin{array}{c} {\dot{M}}_{d}=-K_{d}^{\mathrm{rel}\;}M_{d}+K_{d}^{\text{upt}}\frac{M_{w}}{V}\left(mN_{d}-M_{d}\right) \end{array}.$$

This equation simply expresses the proportionality of the release to the total number of bound drug molecules and the proportionality of the uptake to the total number of free binding sites. Consistent with (16) we complete the set of rate equations corresponding to the scheme in (14)

(17) $$\begin{array}{cc} {\dot{M}}_{w} & =K_{d}^{\mathrm{rel}\;}M_{d}-K_{d}^{\text{upt}}\frac{M_{w}}{V}\left(mN_{d}-M_{d}\right)\\ & \;\;\;\;+K_{a}^{\mathrm{rel}\;}M_{a}-K_{a}^{\text{upt}}\frac{M_{w}}{V}\left(mN_{a}-M_{a}\right),\\ {\dot{M}}_{a} & =-K_{a}^{\mathrm{rel}\;}M_{a}+K_{a}^{\text{upt}}\frac{M_{w}}{V}\left(mN_{a}-M_{a}\right). \end{array}$$

To obtain first-order behavior, we make three assumptions. The first is a steady-state approximation for the number of drug molecules in the aqueous phase, ${\dot{M}}_{w}=0$. The solubility limit of poorly water-soluble drugs is small so that, effectively, any release of drugs from one liposome is accompanied by an immediate uptake by another (or the same [38]) liposome. The second assumption is weak drug loading of all liposomes; this amounts to *M*_*d*_ ≪ *mN*_*d*_, *M*_*a*_ ≪ *mN*_*a*_, and *M* ≪ *mN*. We finally assume the same rate for the uptake of drug molecules from the aqueous phase into donor and acceptor liposomes, implying *K*_*d*_^upt^ = *K*_*a*_^upt^. This is strictly valid only for chemically equivalent donor and acceptor liposomes but should generally be a reasonable approximation. That is, we expect the energy barrier for entering a liposome from the aqueous phase to be small (as compared to the energy barrier for the release from a liposome), irrespective of the liposome's chemical structure. Subject to our three assumptions (16) and (17) become equivalent to

(18) $$\begin{array}{cc} {\dot{M}}_{d} & =-K_{d}^{\mathrm{rel}\;}\frac{N_{a}}{N}M_{d}+K_{a}^{\mathrm{rel}\;}\frac{N_{d}}{N}M_{a},\\ {\dot{M}}_{a} & =K_{d}^{\mathrm{rel}\;}\frac{N_{a}}{N}M_{d}-K_{a}^{\mathrm{rel}\;}\frac{N_{d}}{N}M_{a}. \end{array}$$

Equation (18) are now identical to (6) if we identify *K*_*d*_^*rel*^ = *K*_diff_  (1 − (*kN*_*d*_)/*M*) and *K*_*a*_^*rel*^ = *K*_diff_(1 + *kN*_*a*_/*M*) where *K*_diff_ = *K* appears as the rate constant. Here again, as for (6), the validity of (18) is not subject to a restriction with respect to *N*_*d*_ and *N*_*a*_.

## 3. Discussion

Both transfer mechanisms, through liposome collisions and via diffusion through the aqueous phase, lead to the same first-order kinetic behavior; see (6) and (18). The rate constant of the combined process is

(19) $$\begin{array}{c} K=K_{\text{coll}}\frac{N}{V}+K_{\text{diff}}. \end{array}$$

Its dependence on the *total* liposome concentration allows the experimental determination of the transfer mechanism [13]. We note that the first-order behavior predicted by (6) and (18) requires several assumptions to be fulfilled: low liposome loading with drug molecules, rate constants that are strictly proportional to concentrations of drug molecules, and no intraliposomal kinetics with a rate similar to *K*. In the following, we discuss how the kinetic behavior is predicted to change if any of these assumptions is not fulfilled.

### 3.1. Extension to High Drug Loading

While high drug loading obviously increases the number of available drug molecules (and thus increases the efficiency of liposomal carriers [39]) it also affects the kinetics of the drug release. Our present model predicts such a dependence for the diffusion mechanism whereas the kinetics for the collision mechanism is not affected. Recall that the transition from (16) and (17) to (18) was based on the approximation of weak drug loading, *M*_*d*_ ≪ *mN*_*d*_, *M*_*a*_ ≪ *mN*_*a*_, and *M* ≪ *mN*. Without that approximation, we obtain instead of (18) a nonlinear set of differential equations

(20) $$\begin{array}{cc} {\dot{M}}_{d} & =-K_{d}^{\mathrm{rel}\;}\frac{N_{a}/N-\left(M_{a}/M\right)\left(M/mN\right)}{1-M/mN}M_{d}\\ & \;+K_{a}^{\mathrm{rel}\;}\frac{N_{d}/N-\left(M_{d}/M\right)\left(M/mN\right)\;}{1-M/mN}M_{a},\\ {\dot{M}}_{a} & =K_{d}^{\mathrm{rel}\;}\frac{N_{a}/N-\left(M_{a}/M\right)\left(M/mN\right)}{1-M/mN}M_{d}\\ & \;-K_{a}^{\mathrm{rel}\;}\frac{N_{d}/N-\left(M_{d}/M\right)\left(M/mN\right)}{1-M/mN}M_{a}. \end{array}$$

For the special case that donor and acceptor liposomes are chemically similar, *K*_*d*_^*rel*^ = *K*_*a*_^*rel*^ = *K*_diff_, we obtain a simple exponential behavior

(21) $$\begin{array}{c} M_{a}\left(t\right)=M-M_{d}\left(t\right)=\left(1-{\text{e}}^{-K_{\text{diff}}\;t/\left(1-M/mN\right)}\right)\frac{N_{a}}{N}M \end{array}.$$

**Figure 4:**
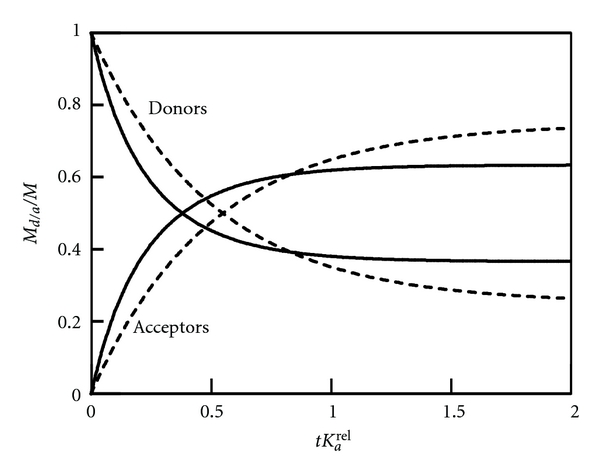
Numerical solutions of (20), derived for *M*/(*Nm*) = 0 (broken lines) and *M*/(*Nm*) = 0.5 (solid lines). The remaining parameters are *K*_*d*_^*rel*^/*K*_*a*_^*rel*^ = 3, *N*_*d*_/*N* = *N*_*a*_/*N* = 0.5. The time *t* is plotted in units of 1/*K*_*a*_^*rel*^.

Here, high drug loading simply increases the rate constant for the diffusion mechanism by the factor 1/(1 − *M*/(*mN*)). In the general case *K*_*d*_^*rel*^ ≠ *K*_*a*_^*rel*^, and no simple exponential decay is predicted for high loading of the liposomes with drug molecules.  Figure 4 shows a numerical example, based on (20) with *K*_*d*_^*rel*^/*K*_*a*_^*rel*^ = 3 and *N*_*d*_/*N* = *N*_*a*_/*N* = 0.5. For *M* ≪ *mN* (weak loading regime; broken lines in Figure 4) we observe the simple exponential behavior according to (18) with equilibrium values *M*_*d*_^eq^/*M* = 1/4 and *M*_*a*_^eq^/*M* = 3/4. For *M*/(*mN*) = 0.5 the initial loading of the donor liposomes is maximal. This leads to both a faster decay and a shift in the equilibrium distribution, reaching $M_{d}^{\text{eq}}/M=(\sqrt{3}-1)/2=0.366$ and $M_{a}^{\text{eq}}/M=(3-\sqrt{3})/2=0.634$. The reason for the increased rate constant is the reduced ability of highly loaded liposomes to take up drug molecules. Hence, if drug molecules are released from initially highly loaded donor liposomes they will be taken up exclusively by acceptor liposomes. The increase in the transfer rate at high loading also affects the equilibrium values *M*_*d*_^eq^/*M* and *M*_*a*_^eq^/*M*. The equilibrium is shifted toward a more uniform distribution of drug molecules between donor and acceptor liposomes (in agreement with Figure 4).

### 3.2. Sigmoidal Behavior

Our model presented so far is unable to predict sigmoidal behavior. That is, no inflection point can be observed in *M*_*d*_(*t*) and *M*_*a*_(*t*). Behind this prediction is our assumption that the transfer rates are strictly proportional to the concentration difference of the drug molecules. For the collision mechanism, this is expressed by our definition of the function *g*(*i*, *j*) in (3). However, if drug molecules within a given liposome interact with each other, the simple relation *g*(*i*, *j*) = *i* − *j* will no longer be valid. More specifically, attractive interactions between drug molecules within liposomes will increase the energy barrier to remove a drug molecule. This becomes relevant at high drug loading. Hence, in the presence of attractive interactions, it will be more unlikely that a drug molecule is transferred from a highly loaded donor liposome to an empty acceptor liposome.

We discuss the consequences of attractive interactions for the collision mechanisms, which is described by (2) and (4). To account for the decrease in the rate constant at high loading we replace (3) by

(22) $$\begin{array}{c} g\left(i,j\right)=\left(i-j\right)\left(1-\frac{i}{m}\right)\left(1-\frac{j}{m}\right) \end{array}.$$

Clearly, for weak loading (*i* ≪ *m* and *j* ≪ *m*) the original first-order model leading to the exponential behavior in (8) is recovered. For large loading of either donor or acceptor liposomes, the transfer rate becomes small. We note that using (22) does not lead to a set of differential equations in terms of only *M*_*d*_(*t*) and *M*_*a*_(*t*). Here, we do not attempt to provide an analytical solution to the problem. Instead, we illustrate its predictions by numerically solving (2) and (4) with *g*(*i*, *j*) given in (22).

**Figure 5:**
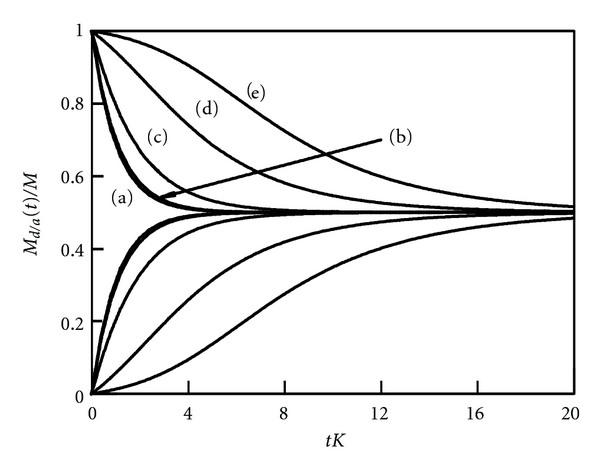
Fraction of drug molecules contained in donor liposomes (*M*_*d*_(*t*)/*M*; upper set of curves) and acceptor liposomes (*M*_*a*_(*t*)/*M*; lower set of curves) as function of the scaled time *Kt*. The curves represent numerical solutions of (2) and (4) with (22), derived for *k* = 0 and *m* = 100 with the initial conditions *d*_*j*_(0) = 0 for *j* ≠ *l*, *d*_*l*_(0) = 100, *a*_*j*_(0) = 0 for *j* > 0, *a*_0_(0) = 100. Different curves correspond to *l* = 2 (a), *l* = 10 (b), *l* = 50 (c), *l* = 90 (d), and *l* = 98 (e).

Figure 5 shows the behavior of *M*_*d*_(*t*) and *M*_*a*_(*t*) as function of *tK* (with *K* = *K*_coll_ *N*/*V*), derived for *m* = 100. For simplicity, we have set *k* = 0 which results in an equipartitioning of drug molecules between donor and acceptor liposomes (*M*_*d*_/*N*_*d*_ = *M*_*a*_/*N*_*a*_ = *M*/*N*). We start with *N*_*d*_ = *N*_*a*_ = 100 liposomes. The acceptor liposomes are initially empty whereas each donor liposome contains initially *l* drug molecules (out of a maximal number *m* = 100). Different curves in Figure 5 correspond to *l* = 2 (a), *l* = 10 (b), *l* = 50 (c), *l* = 90 (d), and *l* = 98 (e). As long as the drug loading is weak (curves (a) and (b)), the solution is simply exponential, characterized by *M*_*a*_/*M* = 1 − *M*_*d*_/*M* = (1 − *e* ^−*Kt*^)*N*_*a*_/*N* (see (8) with *k* = 0). Here, the kinetics is independent of the total number of drug molecules *M* = *lN*_*d*_ (which is why curves (a) and (b) virtually overlap). If the initial loading of the donor liposomes becomes larger (curve (c)) the kinetics slows down. Eventually, once the initial loading approaches its maximal value *mN*_*d*_, the behavior slows down even more and, in addition, becomes sigmoidal. Attractive drug-drug interactions slow down the release from initially highly loaded donor liposomes; at later times (when the donor liposomes are no longer highly loaded), the release becomes faster. This leads to sigmoidal behavior.

### 3.3. Extension to a Two-State Model

In the final part of this work, we briefly discuss an extension of our model to account for two distinct states of the drug molecule inside each liposome. A simple rationale for the presence of two distinct states is provided by the bilayer structure of the liposomes. That is, a drug molecule may preferentially be bound to either the inner or outer monolayer, having to flip-flop in order to change the host monolayer. The typical flip-flop time can be large if the drug has some amphiphilicity or surface activity instead of being strongly lipophilic [40]. Drug molecules residing in the inner monolayer cannot be transported directly to another liposome; they first have to migrate to the outer monolayer.

We denote by *M*_*d*_^*I*^ and *M*_*d*_^*O*^ the number of drug molecules residing in the inner (*D* ^*I*^) and outer (*D* ^*O*^) leaflets of donor liposomes, respectively. Similarly, *M*_*a*_^*I*^ and *M*_*a*_^*O*^ refer to the number of drug molecules residing in the inner (*A* ^*I*^) and outer (*A* ^*O*^) leaflets of acceptor liposomes. The reaction scheme in (10) can then be generalized to account for the inter leaflet transport in donor and acceptor liposomes

(23) $$\begin{array}{c} {\text{D}}^{I}\overset{K_{1}^{d}}{\underset{K_{2}^{d}}{⇌}}{\text{D}}^{O}\overset{K_{1}}{\underset{K_{2}}{\;⇌}}{\text{A}}^{O}\overset{K_{2}^{a}}{\underset{K_{1}^{a}}{⇌}}{\text{A}}^{I} \end{array}.$$

Here, *K*_1_^*d*^ and *K*_2_^*d*^ are the two rate constants corresponding to the transfer of drugs between the two leaflets of the donor liposomes (and similarly for *K*_1_^*a*^ and *K*_2_^*a*^ referring to the acceptor liposomes). The rate constants *K*_1_ = (1 − *kN*_*d*_/*M*)*KN*_*a*_/*N* and *K*_2_ = (1 + *kN*_*a*_/*M*)*KN*_*d*_/*N* are identical to those for the single-state model, where *K* is given in (19). Based on (23), the rate equations can be written as

(24) $$\begin{array}{cc} {\dot{M}}_{d}^{O} & =\frac{K}{N}\left(M_{a}^{O}N_{d}-M_{d}^{O}N_{a}+kN_{a}N_{d}\right)-K_{2}^{d}M_{d}^{O}+K_{1}^{d}M_{d}^{I},\\ {\dot{M}}_{d}^{I} & =K_{2}^{d}M_{d}^{O}-K_{1}^{d}M_{d}^{I},\\ {\dot{M}}_{a}^{O} & =\frac{K}{N}\left(M_{d}^{O}N_{a}-M_{a}^{O}N_{d}-kN_{a}N_{d}\right)-K_{2}^{a}M_{a}^{O}+K_{1}^{a}M_{a}^{I},\\ {\dot{M}}_{a}^{I} & =K_{2}^{a}M_{a}^{O}-K_{1}^{a}M_{a}^{I}. \end{array}$$

In the limit of a symmetric lipid bilayer, the two rate constants for flip-flop of a drug molecule from the inner to the outer leaf and from the outer to the inner leaf are identical (we note that the two leaflets of a liposomal bilayer are not strictly equivalent which, in a more refined model, would entail two different rate constants for flip-flop; this dependence on liposome curvature is neglected here). If we assume furthermore that donor and acceptor liposomes are chemically similar, we may write *K*_1_^*d*^ = *K*_2_^*d*^ = *K*_1_^*a*^ = *K*_2_^*a*^ = *G* as well as *k* = 0. In this case, the rate equations

(25) $$\begin{array}{cc} {\dot{M}}_{d}^{O} & =\frac{K}{N}\left(M_{a}^{O}N_{d}-M_{d}^{O}N_{a}\right)-G\left(M_{d}^{O}-M_{d}^{I}\right),\\ {\dot{M}}_{d}^{I} & =G\left(M_{d}^{O}-M_{d}^{I}\right),\\ {\dot{M}}_{a}^{O} & =\frac{K}{N}\left(M_{d}^{O}N_{a}-M_{a}^{O}N_{d}\right)-G\left(M_{a}^{O}-M_{a}^{I}\right),\\ {\dot{M}}_{a}^{I} & =G\left(M_{a}^{O}-M_{a}^{I}\right) \end{array}$$

depend on only two parameters, the rate constants *K* and *G*. If we assume all drug molecules initially reside in the donor liposomes, the initial conditions are *M*_*d*_^*O*^(*t* = 0) = *M*_*d*_^*I*^(*t* = 0) = *M*/2, and *M*_*a*_^*O*^(*t* = 0) = *M*_*a*_^*I*^(*t* = 0) = 0, where *M* is the total number of drug molecules in the system. The solution of (25) can be expressed as

(26) $$\begin{array}{cc} M_{d}^{I}\left(t\right) & =\frac{M}{2}\left[\frac{N_{d}}{N}+\frac{N_{a}}{N}\;\frac{\omega_{2}e^{-\omega_{1}t}-\omega_{1}e^{-\omega_{2}t}}{\omega_{2}-\omega_{1}}\right],\\ M_{d}^{O}\left(t\right)-M_{d}^{I}\left(t\right) & =\frac{M}{2}K\frac{N_{a}}{N}\;\frac{e^{-\omega_{2}t}-e^{-\omega_{1}t}}{\omega_{2}-\omega_{1}},\\ M_{a}^{I}\left(t\right) & =\frac{MN_{a}}{2N}\left[1-\frac{\omega_{2}e^{-\omega_{1}t}-\omega_{1}e^{-\omega_{2}t}}{\omega_{2}-\omega_{1}}\right],\\ M_{a}^{O}\left(t\right)-M_{a}^{I}\left(t\right) & =\frac{M}{2}K\frac{N_{a}}{N}\frac{e^{-\omega_{1}t}-e^{-\omega_{2}t}}{\omega_{2}-\omega_{1}}. \end{array}$$

The solution is thus a combination of exponential decays with corresponding effective rate constants *ω*_1_ and *ω*_2_. Such biexponential behavior has been observed for the spontaneous transfer of certain lipids between phosphatidylcholine vesicles [41] and also for the release behavior of an imidazole derivate from liposomes [42]. The effective rate constants *ω*_1_ and *ω*_2_ can be calculated from *G* and *K* through

(27) $$\begin{array}{c} 2G+K=\omega_{1}+\omega_{2},\;\;4G^{2}+K^{2}={\left(\omega_{2}-\omega_{1}\right)}^{2}. \end{array}$$

**Figure 6:**
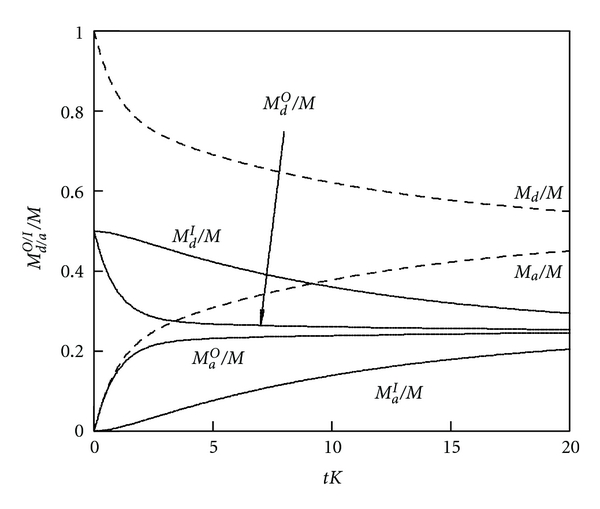
Fractions of drug molecules in inner and outer leaflets of donor and acceptor liposomes. The quantities *M*_*d*_^*O*^(*t*), *M*_*d*_^*I*^(*t*), *M*_*a*_^*O*^(*t*), and *M*_*a*_^*I*^(*t*) are plotted according to (26) for *G*/*K* = 1/10 and *N*_*a*_/*N* = *N*_*d*_/*N* = 0.5. The broken lines show the biexponential behaviors of the sums *M*_*d*_ = *M*_*d*_^*O*^ + *M*_*d*_^*I*^ and *M*_*a*_ = *M*_*a*_^*O*^ + *M*_*a*_^*I*^. The time is plotted in units of the inverse rate constant *K*. Note also *ω*_1_ = 1.11*K* and *ω*_2_ = 0.09 *K* are the effective rate constants for the decay.

Hence, a measurement of *ω*_1_ and *ω*_2_ could be used to obtain the two model parameters (*K* and *G*).  Figure 6 displays a plot of *M*_*d*_^*O*^(*t*), *M*_*d*_^*I*^(*t*), *M*_*a*_^*O*^(*t*), *M*_*a*_^*I*^(*t*), *M*_*a*_(*t*), *M*_*d*_(*t*), calculated for *G*/*K* = 1/10 and *N*_*a*_/*N* = *N*_*d*_/*N* = 0.5. All drug molecules are initially distributed equally among the two leaflets of the donor liposomes. Release of drug molecules from the outer leaf of the donor liposomes is fast (*K* = 10*G*), the slow process is the flip-flop of drug molecules between the two leaflets of the liposomes. Hence, at intermediate times, say at *t* = 3/*K*, the outer leaflets have almost reached their equilibrium values whereas the inner layers remain still fairly close to their initial values. After reaching thermal equilibrium (*t* → *∞*), half of all drug molecules have migrated to the acceptor liposomes. Clearly, the presence of the two different rate constants (*K* and *G*) leads to the biexponential behavior of *M*_*d*_ and *M*_*a*_ in Figure 6.

We briefly discuss two limiting cases for (26). First, for *G* = 0 the flip-flop of drug molecules between the inner and outer leaves is infinitely slow, implying *M*_*d*_^*I*^(*t*) = *M*/2, *M*_*a*_^*I*^(*t*) = 0, *M*_*a*_^*O*^(*t*) = *M*/2 − *M*_*d*_^*O*^(*t*) = (1 − *e* ^−*Kt*^)(*MN*_*a*_)/(2*N*). In this case, we recover the kinetics of (8), yet with only *M*/2 (instead of *M*) drug molecules participating in the transfer and identical donor and acceptor liposomes (*k* = 0). Second, for *G* → *∞* flip-flop becomes infinitely fast and (26) read *M*_*a*_^*I*^(*t*) = *M*_*a*_^*O*^(*t*) = *M*/2 − *M*_*d*_^*I*^(*t*) = *M*/2 − *M*_*d*_^*O*^(*t*) = (1 − *e* ^−*Kt*/2^)(*MN*_*a*_)/(2*N*). Because 50% of the drug molecules reside in the inner leaflets, they do not contribute to the outer-leaflet-concentration-differences that drive the transfer kinetics. Hence, the apparent rate constant is reduced from *K* to *K*/2.

## 4. Conclusions

In this work, we have presented a detailed model for the transfer kinetics of poorly water-soluble drug molecules between liposomal carrier systems. Apart from liposomes, the scope of the model includes other types of small and mobile pharmaceutical nanocarriers, such as micelles, colloids, and nanoparticles. Starting from a microscopic distribution function of drug molecules among donor and acceptor liposomes, we have specified the conditions that lead to an apparent first-order kinetic behavior. These include low drug loading of the liposomes, strict proportionality of all rate constants to drug concentrations, no aggregation phenomena of drugs within liposomes, and no overlap of the intraliposomal flip-flop kinetics. Systems that do not fulfill these conditions do not, generally, exhibit an apparent first-order kinetics. Instead the behavior may become biexponential or sigmoidal. High drug loading may preserve the first order kinetics but with increased apparent rate constant.

An optimal drug delivery system should keep the drug load on the way to the target and release it only after arrival at the target. Understanding the kinetics and mechanisms of drug release from liposomal (and other) nanocarriers is thus a prerequisite to systematically improving drug delivery systems.
